# Supplementary material for: Hypercapnia as a Double-Edged Modulator of Innate Immunity and Alveolar Epithelial Repair: A PRISMA-ScR Scoping Review
Source: Int J Mol Sci. 2025 Oct 2;26(19):9622. doi: 10.3390/ijms26199622 (PMC12524586; doi:10.3390/ijms26199622)
Supplement: Supplementary file 1 [file ijms-26-09622-s001.zip › ijms-3560799-supplementary.pdf]

International Journal of
*Molecular Sciences*

MDPI

Supplementary Material

# Hypercapnia as a Double-Edged modulator of Innate Immunity and Alveolar Epithelial Repair: A PRISMA-ScR Scoping Review

## Materials and Methods

*1.1. Operational definitions & coding rules*

- Primary exposure metric: $PaCO_2$ in mmHg; $\%FiCO_2$ kept descriptive when arterial $PaCO_2$ not reported.
- Buffered vs unbuffered: "Buffered" if authors state pH control (7.35–7.45) or explicit buffering; "Unbuffered" if acidemia permitted; "pH n.s." if not specified.
- Binning (heat maps): $PaCO_2$ and time bins as in Figure 2 legend.
- Effect scoring: per Table S1 hierarchy.
- Multi-arm studies: each arm captured separately; one study can contribute to multiple cells; within-cell averaging applied.
- Infectious label: live pathogen exposure = infectious; LPS/TLR-only = non-infectious (noted as "infection-mimic").
- Handling missingness: no imputation; cells remain blank if no data.

*1.2. Sensitivity analyses*

To assess the specificity of hypercapnia's effects, we contrasted effect direction against predefined combinations of context (infectious vs. non-infectious), dose–time bins ($PaCO_2$ and exposure duration), and acid–base status (buffered vs. unbuffered), and examined concordance with mechanistic axes. The protective signal was largely confined to non-infectious models with moderate $PaCO_2$ (~65–95 mmHg) and brief exposures (≤4–6 h), where attenuation of canonical NF-κB, cytokine down-modulation, and preservation of epithelial ion transport co-occurred. In contrast, the harmful signal clustered specifically in infectious settings and/or with prolonged exposures (≥24–96 h) or elevated $PaCO_2$ (≥110–120 mmHg), featuring higher bacterial burden and epithelial dysfunction (endocytosis of ENaC/Na,K-ATPase via AMPK/PKA/ERK1/2), together with an immunosuppressive bias (non-canonical RelB/p52 activation and inhibition of autophagy through the Bcl-2/Bcl-xL–Beclin-1 axis). These patterns persisted in sensitivity analyses (excluding $\%CO_2$-only studies, shifting bin boundaries by ±10 mmHg, and excluding exposures <30 min or >120 h), supporting that the association is context- and exposure-specific rather than an aggregation artifact. Isolated exceptions were explained by differences in pH status (buffered vs. unbuffered), antibiotic control, and cell type, further reinforcing the biological validity of the proposed specificity map.

**TABLES**

**Table S1.** Decision log (post-hoc protocol clarifications).

| Decision | Rationale | Impact on analysis/reporting |
|---|---|---|
| Register protocol on OSF after defining time window (DOI: 10.17605/OSF.IO/WV85T; 30-Aug-2024) | Ensure transparency despite late registration | Marked as post-hoc in Methods; all subsequent changes recorded below |
| Prefer $PaCO_2$ (mmHg) over $\%FiCO_2$ | Physiologic comparability across models | Medians/IQR computed only from $PaCO_2$; studies with $\%FiCO_2$ alone labelled "$\%CO_2$ only" and excluded from $PaCO_2$ summaries |
| Buffered vs unbuffered classification | Acid–base state modulates biology | "Buffered" if authors report pH 7.35–7.45 or explicit buffering (e.g., $HCO_3^-$/HEPES); otherwise "Unbuffered" or "pH n.s."; used for subgroup comments, not exclusion |
| Acute vs chronic windows | Different biology over time | Acute = 0–120 h (heat maps); Chronic = >120 h (described narratively only) |
| Effect-direction scoring | Needed a unified signal across heterogeneous endpoints | +1 protective; 0 mixed/neutral; −1 harmful. Endpoint hierarchy: (1) lung mechanics/histology, (2) bacterial load, (3) epithelial transport: ENaC/Na,K-ATPase/**AFC**, (4) phagocytosis/autophagy, (5) cytokines, (6) transcriptional changes. If endpoints conflict within a study arm → 0 (mixed) |
| Cell averaging in heat maps | Multiple arms fall into same bin | When multiple data points share a cell, average their scores (e.g., +1 and 0 → +0.5; rendered as intermediate color) |
| Infectious vs non-infectious label | Consistent context stratification | Live pathogen = infectious; LPS/TLR-only = non-infectious (infection-mimic) for innate outputs |
| Handling missing exposure data | Avoiding spurious conversions | No back-calculation of $PaCO_2$ from $\%FiCO_2$; when only $\%FiCO_2$ is available, keep descriptive tag "$\%CO_2$ only" |
| Time & $PaCO_2$ bin edges | Balance interpretability and data density | $PaCO_2$: 40–<60; 60–<80; 80–<100; 100–<120; ≥120 mmHg. Time: 0–≤1; >1–≤4; >4–≤6; >6–≤24; >24–≤96; >96–≤120 h |
| Sensitivity analyses (pre-specified) | Robustness check | (i) Exclude "$\%CO_2$ only"; (ii) shift $PaCO_2$ bins ±10 mmHg; (iii) treat within-study conflicts as fractional instead of 0; (iv) in vivo only; (v) exclude <30 min and >120 h |
| Software & figure generation | Reproducibility | Descriptives in jamovi 2.2.5; heat maps rendered with a diverging scale (−1 red, 0 grey, +1 green); scripts deposited in OSF |
| Risk-of-bias | Scoping review by design | No formal SYRCLE; design domains extracted and discussed in Limitations |

Abbreviations: AFC: Alveolar Fluid Clearance; ENaC: Epithelial Sodium Channels; LPS: Bacterial Lipopolysaccharide; N.S.: Not Significant; TLR: Toll-Like Receptor.

**Table S2.** Preferred Reporting Items for Systematic reviews and Meta-Analyses extension for Scoping Reviews (PRISMA-ScR) Checklist.

| SECTION | ITEM | PRISMA-ScR CHECKLIST ITEM | REPORTED ON PAGE # |
|---|---|---|---|
| **TITLE** | | | |
| Title | 1 | Identify the report as a scoping review. | 1 |
| **ABSTRACT** | | | |
| Structured summary | 2 | Provide a structured summary that includes (as applicable): background, objectives, eligibility criteria, sources of evidence, charting methods, results, and conclusions that relate to the review questions and objectives. | 1 |
| **INTRODUCTION** | | | |
| Rationale | 3 | Describe the rationale for the review in the context of what is already known. Explain why the review questions/objectives lend themselves to a scoping review approach. | 1,2 |
| Objectives | 4 | Provide an explicit statement of the questions and objectives being addressed with reference to their key elements (e.g., population or participants, concepts, and context) or other relevant key elements used to conceptualize the review questions and/or objectives. | 2 |
| **METHODS** | | | |
| Protocol and registration | 5 | Indicate whether a review protocol exists; state if and where it can be accessed (e.g., a Web address); and if available, provide registration information, including the registration number. | 2 |
| Eligibility criteria | 6 | Specify characteristics of the sources of evidence used as eligibility criteria (e.g., years considered, language, and publication status), and provide a rationale. | 2 |
| Information sources* | 7 | Describe all information sources in the search (e.g., databases with dates of coverage and contact with authors to identify additional sources), as well as the date the most recent search was executed. | 3 |
| Search | 8 | Present the full electronic search strategy for at least 1 database, including any limits used, such that it could be repeated. | 2, Appendix A1 |
| Selection of sources of evidence† | 9 | State the process for selecting sources of evidence (i.e., screening and eligibility) included in the scoping review. | 3 |
| Data charting process‡ | 10 | Describe the methods of charting data from the included sources of evidence (e.g., calibrated forms or forms that have been tested by the team before their use, and whether data charting was done independently or in duplicate) and any processes for obtaining and confirming data from investigators. | 3, Figure 1, PRISMA-ScR checklist |
| Data items | 11 | List and define all variables for which data were sought and any assumptions and simplifications made. | 3 |
| Critical appraisal of individual sources of evidence§ | 12 | If done, provide a rationale for conducting a critical appraisal of included sources of evidence; describe the methods used and how this information was used in any data synthesis (if appropriate). | 3 |
| Synthesis of results | 13 | Describe the methods of handling and summarizing the data that were charted. | 3 |
| **RESULTS** | | | |
| Selection of sources of evidence | 14 | Give numbers of sources of evidence screened, assessed for eligibility, and included in the review, with reasons for exclusions at each stage, ideally using a flow diagram. | Figure 1, PRISMA-ScR checklist |
| Characteristics of sources of evidence | 15 | For each source of evidence, present characteristics for which data were charted and provide the citations. | Table 2 |
| Critical appraisal within sources of evidence | 16 | If done, present data on critical appraisal of included sources of evidence (see item 12). | |
| Results of individual sources of evidence | 17 | For each included source of evidence, present the relevant data that were charted that relate to the review questions and objectives. | Table 2, Figure 2, 3, and 4 |
| Synthesis of results | 18 | Summarize and/or present the charting results as they relate to the review questions and objectives. | Table 2 |
| **DISCUSSION** | | | |

| SECTION | ITEM | PRISMA-ScR CHECKLIST ITEM | REPORTED ON PAGE # |
|---|---|---|---|
| Summary of evidence | 19 | Summarize the main results (including an overview of concepts, themes, and types of evidence available), link to the review questions and objectives, and consider the relevance to key groups. | 11 – 15 |
| Limitations | 20 | Discuss the limitations of the scoping review process. | 15 |
| Conclusions | 21 | Provide a general interpretation of the results with respect to the review questions and objectives, as well as potential implications and/or next steps. | 16 |
| **FUNDING** | | | |
| Funding | 22 | Describe sources of funding for the included sources of evidence, as well as sources of funding for the scoping review. Describe the role of the funders of the scoping review. | **NA** |

JBI = Joanna Briggs Institute; PRISMA-ScR = Preferred Reporting Items for Systematic reviews and Meta-Analyses extension for Scoping Reviews.

* Where *sources of evidence* (see second footnote) are compiled from, such as bibliographic databases, social media platforms, and Web sites.

† A more inclusive/heterogeneous term used to account for the different types of evidence or data sources (e.g., quantitative and/or qualitative research, expert opinion, and policy documents) that may be eligible in a scoping review as opposed to only studies. This is not to be confused with *information sources* (see first footnote).

‡ The frameworks by Arksey and O'Malley (6) and Levac and colleagues (7) and the JBI guidance (4, 5) refer to the process of data extraction in a scoping review as data charting.

§ The process of systematically examining research evidence to assess its validity, results, and relevance before using it to inform a decision. This term is used for items 12 and 19 instead of "risk of bias" (which is more applicable to systematic reviews of interventions) to include and acknowledge the various sources of evidence that may be used in a scoping review (e.g., quantitative and/or qualitative research, expert opinion, and policy document).

*From:* Tricco AC, Lillie E, Zarin W, O'Brien KK, Colquhoun H, Levac D, et al. PRISMA Extension for Scoping Reviews (PRISMAScR): Checklist and Explanation. Ann Intern Med. 2018;169:467–473. doi: 10.7326/M18-0850.
